# Increased Risk of Coronary Artery Disease in People with Diagnosis of Neuromuscular Disorders: A Nationwide Retrospective Population-Based Case–Control Study

**DOI:** 10.3390/diagnostics14020199

**Published:** 2024-01-16

**Authors:** Yi-Chuan Chang, Ing-Shiow Lay, Cheng-Hao Tu, Yu-Chen Lee

**Affiliations:** 1 Graduate Institute of Acupuncture Science, College of Chinese Medicine, China Medical University, Taichung 404328, Taiwan; 065715@tool.caaumed.org.tw; 2 Department of Chinese Medicine, China Medical University Beigang Hospital, Yunlin 651012, Taiwan; 061540@tool.caaumed.org.tw; 3 School of Post-Baccalaureate Chinese Medicine, China Medical University, Taichung 404328, Taiwan; 4 School of Chinese Medicine, China Medical University, Taichung 404328, Taiwan; 5 Department of Chinese Medicine, China Medical University Hospital, Taichung 404328, Taiwan

**Keywords:** coronary artery disease, carpal tunnel syndrome, stenosing tenosynovitis, medial epicondylitis, ulnar nerve injury

## Abstract

The existing literature has explored carpal tunnel syndrome (CTS) and determined that it could be a risk for coronary artery disease (CAD), but there has been little research comparing the relevance of CAD with other neuromuscular disorders (NMDs) to CTS. This case–control study explored the association between CTS, stenosing tenosynovitis (ST), and ulnar side NMDs and CAD. The study utilized data from Taiwan’s National Health Insurance Research Database, focusing on health insurance claims. Between January 2000 and December 2011, we employed the International Classification of Diseases, Ninth Revision, Clinical Modification (ICD-9-CM) diagnostic codes to identify 64,025 CAD patients as the case group. The control group consisted of an equal number of individuals without CAD, matched for age, sex, and index year of CAD. Logistic regression analysis was employed to calculate the odds ratios (ORs) and 95% confidence intervals (CIs) for each variable. Multivariate analysis, after adjusting for sociodemographic factors and comorbidities, revealed a significantly higher likelihood of a previous diagnosis of CTS in the CAD group compared to the comparison control group. However, neither ST nor the ulnar side NMDs had any statistical significance. These results indicated that median nerve injury, rather than other NMDs, may uniquely serve as a predisposing factor of CAD.

## 1. Introduction

Coronary artery disease (CAD), which is the most common of the cardiovascular diseases (CVDs), has been found to be the leading cause of death in both developed and developing countries [1]. The typical symptoms include chest pain; substernal discomfort; heaviness; a pressure-like feeling or discomfort which may travel into the shoulder, arm, back, neck, or jaw; and shortness of breath [2]. Other complications, such as palpitations, could be self-awareness, especially in acute coronary syndrome patients [3]. The symptoms may be aggravated by exercise, emotional stress, or relief after rest [2], but sometimes there could be no symptoms existing in daily life, or they may not be found until sudden cardiac death. Although there are different types and symptoms of CAD [4], the main cause of the pathological changes is a thickening of the inside walls of the coronary arteries, which is known as atherosclerosis, leading to narrowing of the vessel lumina, a decrease in blood flow, obstruction of the oxygen supply, and an exchange of metabolism, which may cause the symptoms listed above or even decease eventually.

The risk factors associated with CAD have been established by numerous and extensive epidemiological study results. The major risk factors are hypertension (HTN), diabetes mellitus (DM), hyperlipidemia, and smoking [2,5,6,7]; the second most important factors are obesity, hyperuricemia, physiological, and psychosocial stress [2]. Moreover, the prevalence of CAD markedly increases with age [8] and is higher in males than females in almost all stages of different ages [9]. However, some literature indicated that patients with chronic inflammatory muscle diseases may also have higher rates of CADs with increased permeability of endothelial cells to inflammatory cells, with subendothelial lipid deposition, oxidation, and progression of coronary atherosclerosis [10]. One cohort design study also reported that there is a strong relation between lateral epicondylitis and CVD risk factors, along with a potentially modifiable disease mechanism [11]. Similar conclusions were also reported in the research which mentioned glenohumeral joint pain, which demonstrates a strong correlation between CVD risk factors and rotator cuff tendinopathy [12]. A recent publication also suggested that a modified Framingham CVD risk score is strongly related to both carpal tunnel syndrome (CTS) diagnosis and an abnormal median nerve conduction study, with odds ratios that were over fourfold and sevenfold higher [13]. In our previous case–control study of data analysis, we also found a significantly increased risk between CAD and a previous diagnosis of CTS in the case group compared to the comparison control group (*p* < 0.0001) [14].

The aforementioned recent studies indicated that some neuromuscular disorders (NMDs) may exist that are relevant to CAD/CVD or that share similar risk factors, especially with CTS. But the relevance between CAD and other NMDs is still not clear. Hence, the purpose of this study extends from our previous study and provides further analysis to investigate the association between CAD and NMDs in the nearby region of the forearm. We hypothesize that the correlation between CAD and CTS could be relatively strong and relatively weak or that there could be no statistical difference between CAD and other NMDs.

## 2. Materials and Methods

### 2.1. Data Resources

This case–control study used data from the Longitudinal Health Insurance Database 2000 (LHID2000), a subset of Taiwan’s National Health Insurance Research Database (NHIRD). The NHIRD database covers records for more than 99% of the total population; it is derived from reimbursement claims covering all of Taiwan, and it contains all-embracing data, such as basic demographic information, clinical visits and hospitalization records, diagnosis and assessment results, procedures and prescriptions delivered, and medical costs for reimbursement.

In this study, for personal data protection to prevent any possible identification of the subjects, the entire dataset was de-identified and encrypted before being made available for analysis and publication. The diseases, such as CAD and CTS, contained in the study for analyses were identified by the International Classification of Diseases, Ninth Revision, Clinical Modification (ICD-9-CM) diagnostic codes, and each diagnosis needed to be referenced a minimum of three times on separate occasions for confirmation.

### 2.2. Study Subjects and Study Variables

Building on our prior research, we randomly selected 64,025 subjects newly diagnosed with CAD (ICD-9-CM codes 410-414) as the case group and 64,025 individuals without CAD as controls from the same NHIRD database. The controls were frequency-matched to the cases by age (within 5 years), sex, and index year of CAD. The index year of CAD for each case was defined as the year of the initial diagnosis, while for the controls, it was randomly assigned between January 2000 and December 2011 and aligned with the index year of the matched CAD case. In this study, all the participants were screened between January 2000 and the end of 2011 and subsequently monitored until their death, or the onset of a CTS (ICD-9-CM codes 354.0, 354.1), stenosing tenosynovitis (ST; ICD-9-CM code 727.04), or ulnar side NMDs, including medial epicondylitis (ICD-9-CM code 726.31), ulnar nerve lesion (ICD-9-CM code 354.2), or ulnar nerve injury (ICD-9-CM codes 955.2), all of which were previously diagnosed in all the participants for intergroup comparison at the individual level, to evaluate their relevance to CAD.

**Figure 1:**
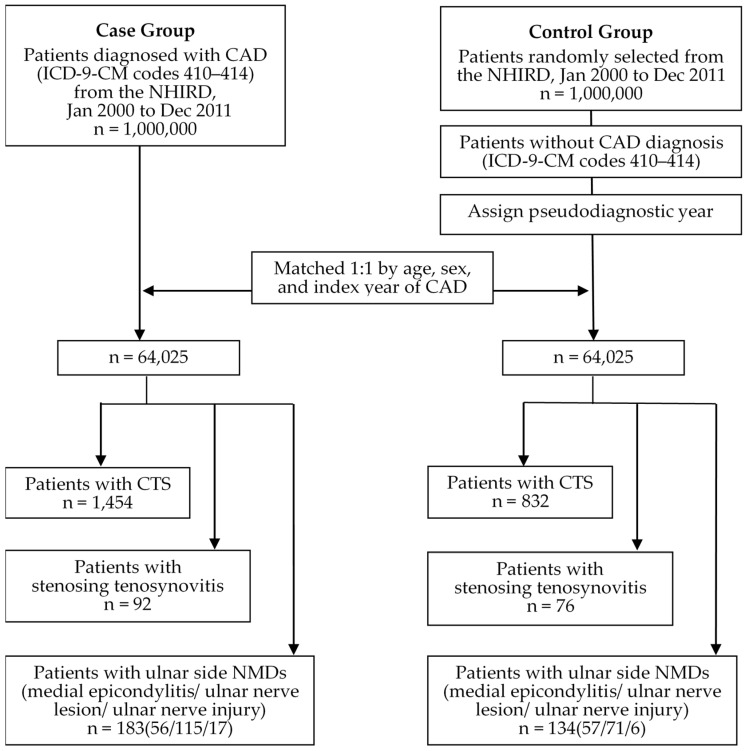
Flow chart of subjects from the LHID 2000 for patients with/without CAD as case/control group.

The patients with documented histories of DM (ICD-9-CM code 250), HTN (ICD-9-CM codes 401–405), or hyperlipidemia (ICD-9-CM code 272) at the index date were classified as having comorbidities with CAD. All the diagnosis codes above also had to be cited at least three or more times on different dates for confirmation. All of the stages of the study flow chart are presented in Figure 1.

### 2.3. Study Subjects and Study Variables

The statistical analyses utilized SAS^®^ software, version 9.4 (SAS Institute Inc., Cary, NC, USA). Descriptive statistics were used to compare the baseline characteristics between the cases and controls. Categorical variables were assessed using Pearson’s chi-squared test, and continuous variables were analyzed using the Student’s *t*-test. Logistic regression estimated the odds ratios (ORs) and 95% confidence intervals (CIs). Multivariate analysis adjusted for sociodemographic factors and comorbidities. The data were presented as the mean with standard deviation (SD) or rates with 95% CIs. Statistical significance was set at *p* < 0.05.

## 3. Results

The demographic and clinical characteristics of the study population are presented in Table 1. Both the case group (mean ± SD: 60.84 ± 13.40 years old) and the control group (mean ± SD: 59.91 ± 13.70 years old) included 64,025 subjects, and the sex and age distributions were similar between the groups. The proportion of male subjects was slightly higher than that of the female subjects (52.01% to 47.99%), and most of the patients were aged over 40 years. In the case group, 1454 (2.27%) of the patients had a previous diagnosis of CTS, 92 (0.14%) of the patients had a previous diagnosis of ST, and 183 (0.29%) of the patients had a previous diagnosis of ulnar side NMDs; of the controls, 832 (1.3%) patients had a prior diagnosis of CTS, 76 (0.12%) of the patients had a previous diagnosis of ST, and 134 (0.21%) of the patients had a previous diagnosis of ulnar side NMDs. Compared with the controls, the comorbidity risk factors, including DM, hypertension, and hyperlipidemia, were highly statistically significant between the two groups.

The odds ratios for the study population are presented in Table 2. The adjusted ORs are the results after adjusting for potentially confounding variables in the logistic regression modeling. The CAD group exhibits a significantly higher likelihood of CTS diagnoses than the controls in either the crude (crude OR: 1.77, 95% CI 1.62–1.92; *p* < 0.0001) or adjusted (adjusted OR: 1.46, 95% CI 1.32–1.59; *p* < 0.0001) statistical results. However, this association does not persist in the ST or ulnar side NMDs diagnoses. In neither the crude (crude OR: 1.21, 95% CI 0.89–1.64; *p* = 0.2188) nor the adjusted (adjusted OR: 1.12, 95% CI 0.8–1.55; *p* = 0.5195) statistical results of the ST diagnoses were there existing significant differences in the CAD group compared to the controls. Then, despite the statistical difference (crude OR: 1.36, 95% CI 1.09–1.70; *p* = 0.0065) in the results of the crude OR analysis of ulnar sided NMDs, the differences did not exist after being adjusted (adjusted OR: 1.10, 95% CI 0.87–1.41; *p* = 0.4277).

We further employed the same analytical approach to formulate a cohort study analysis to assess the hazard ratios (HRs) between CTS and CAD, wherein we systematically aligned groups afflicted with CTS within the database, as shown in Table 3. This strategic alignment aimed to bolster the robustness of the aforementioned findings. By excluding subjects with documented CAD predating the onset of CTS, we identified a cohort group of 6188 subjects (mean ± SD: 46.22 ± 10.8 years old) and the control group (mean ± SD: 46.14 ± 10.89 years old). Subsequently, our analysis revealed that 508 cases in the cohort group (8.21%) and 282 cases in the control group (4.56%) eventually manifested CAD. The tabulated analysis results also elucidate the fact that, irrespective of whether it is the cohort group grappling with CTS or the control group, a higher incidence of future CAD is discernable among the female subjects. With the exception of individuals under 20 years old, the incidence of CAD in the cohort group exceeded that of the control group. Concurrently, in terms of comorbidities, the prevalence of HTN, DM, and hyperlipidemia in the cohort group also surpassed that observed in the control group.

Within the HR results section conducted in the cohort study mode, the adjusted hazard ratios (aHRs) represent the outcomes subsequent to the adjustment for potential confounding variables in the Cox proportional hazards regression. In comparison to the non-CTS group, the CTS cohort group demonstrated an elevated risk of developing CAD (aHR = 1.61; 95% CI = 1.39–1.87, *p* < 0.001) following adjustments for CTS, age, gender, and comorbidities. In the context of the secondary analysis outcomes concerning various variables, it is imperative to note that this result remains amenable to validation across diverse genders and age groups. With the exception of the absence of subjects afflicted by CAD in the subgroups aged below 20 years in both groups, no statistically significant distinctions were discerned between the two groups in the subgroups comprising individuals over 60 years old, as evidenced by the outcomes derived from both the crude and adjusted HR analyses. This finding aligns consistently with the aforementioned results in NMDs analysis, suggesting a discernible correlation between CTS and the occurrence of CAD.

## 4. Discussion

This study explores the risk of CAD in individuals with a prior CTS diagnosis and evaluates the strength of the association between CAD and other NMDs. The result of our NHIRD data analysis is consistent with our hypothesis that patients with a previous diagnosis of CTS are significantly more likely to have concomitant CAD (*p* < 0.0001), whereas no such significant statistic association was identified between CAD and the other NMDs, such as ST or medial epicondylitis/ulnar nerve lesion/ulnar nerve injury after adjusting the univariate and multivariate logistic regression analyses.

### 4.1. The Possible Linking between NMDs and Coronary Artery Diseases

The study results revealed that some of the NMDs, such as ulnar nerve injury, posed a significant risk factor for CAD in the unadjusted model; however, it was found to be insignificant after adjusting for comorbidity factors. This suggests that the risk of NMDs for CAD may be caused by similar risk factors that lead to CAD. Specifically, these results suggest that NMD does not directly cause CAD; rather, DM, HTN, and hyperlipidemia, which are comorbid with both NMD and CAD, may simultaneously elevate the risk of both conditions. As a result, the significant risk observed in the unadjusted model disappears after controlling for these confounding variables. The association of metabolic disorders and risk factors for atherosclerosis with NMDs has recently received increasing discussion and attention to varying degrees [15,16]. In contrast, as in our prior research [14], the present study findings indicate that CTS continues to pose a significant risk factor for CAD even after adjusting for comorbidity factors. This suggests that CTS may directly contribute to the development of CAD, or both conditions may share additional common risk factors apart from DM, HTN, and hyperlipidemia.

Obesity, as indicated by a high body mass index (BMI), is a potential common risk factor between CTS and CAD. Although several established risk factors for CTS exist, such as smoking [17,18,19], diabetes mellitus [20,21], low-density lipoprotein (LDL) cholesterol [22], and obesity [21,23,24], it should be noted that these factors are also consistent with those associated with atherosclerosis [10]. Moreover, in addition to the positive correlation observed between body mass index (BMI) and the long-term prognosis of CAD [25], in metabolic syndrome higher BMI, rather than waist circumference or waist-to-hip ratio, and LDL were determined as risk factors correlated with the severity of CTS [26,27,28]; each 1-unit rise in BMI raises the risk of CTS by 7.4% [29]. However, the evidence associated with workload exposure and CTS is fluctuant and contradictory [30], and the prevalence levels may also vary across the different occupations and industries [23,30]. Regarding the possible pathological link between obesity and CTS, Otelea et al. proposed the following three possible mechanisms [31]: (1) the direct compression of the fat deposition around the wrist; (2) the insulin resistance, dyslipidemia, and inflammatory and oxidative mechanisms, which affect the peripheral nervous system indirectly through vascular and muscle and tendon impairment and are related to the central deposition of the fat; and (3) the impaired muscle contraction and metabolism related to myosteatosis. The scholars also believe that reducing obesity will not only improve cardio-metabolic health but also reduce the occurrence and prognosis of CTS [31].

Additional factors that may potentially link CTS to CAD include oxidative stress and carotid intima-media thickness (IMT). It has been reported that oxidative stress and decreased antioxidant capacity may lead to fibrosis through a disturbed signaling pattern in the tenosynovium and median nerve, leading to CTS [32]. There are also many mechanisms and publications confirming oxidative stress and the formation of CAD [33,34]. Carotid IMT could also be regarded as a pathological index to CTS in the scientific reports that build a bridge between CAD and CTS [35,36,37]. Furthermore, study results also determined that there is not only a higher prevalence of CTS among CAD groups but also other CVDs, such as valvular heart disease [36]. These reports expose the possible underlying mechanisms and pathological changes associated with CTS and CAD, including obesity and other associated metabolic issues, oxidative stress, and chronic inflammation, and the pathological changes between CTS and cardiovascular risk factors appear to be associated with increased carotid IMT [35]. CTS may be a manifestation of atherosclerosis beyond the traditional cardiovascular risk factors that affects the occurrence of CAD progressively [36]. Similarly, elderly men with transthyretin (TTR)-related amyloidosis exhibit a higher adjusted prevalence of CTS compared to the general population. CTS can be considered a prognostic marker in TTR-related amyloidosis and an additional risk factor for cardiac amyloidosis, irrespective of cardiac involvement [38]. Recognizing this association introduces the prospect of an alternative perspective on the connection between CTS and CAD.

Alternatively, considering the physiological perspective, within the recent publications from our team the investigations have revealed that the stimulation of the median nerve at various intensities has the capacity to modulate the parameters associated with heart rate variability (HRV) [39]. These parameters encompass the activity of the parasympathetic (PNS) and sympathetic nervous systems (SNS), and the association with CAD is also attributed to abnormal performance or heightened activity of the SNS [40]. In focusing the analysis on the brain regions responsible for regulating the PNS or SNS, such as the rostral ventrolateral medulla, it becomes evident that varying intensities of stimulation yield not only divergent effects on the parameters associated with HRV but that distinct functional connectivity changes are also observable in other areas of the brain [41]. This intriguing association between nerve stimulation and physiological responses may offer a nuanced perspective, potentially serving as a rational foundation for understanding the enduring link between CTS and CAD.

### 4.2. NHIRD, the Applications of Big Data Analysis

The utilization of big data analytics in medical engineering and healthcare is increasingly gaining attention. It has been observed that stakeholders are adopting big data analytics to minimize medical costs and personalize medical services for each patient, resulting in more effective healthcare management [42]. NHIRD is a database that is exclusive to Taiwan. NHIRD data analyses empower researchers to explore associations between diseases, to monitor clinical disease progression, and to investigate the outcomes of diverse medical interventions. This database provides a large depository of clinical records for researchers to speculate on as many kinds of original, innovative research approaches as possible. The application includes risk factor evaluation and analysis, such as the exploration of the risks to the mental states of nurses [43]; the association between Parkinson’s disease and proton pump inhibitor use [44]; the possible links between diseases, like the increased risk of Bell’s palsy in patients with migraine [45]; the relationship between Alzheimer’s disease and chronic periodontitis [46]; or the higher incidence of leukemia in the hyperuricemia adults [47]. Our study delves into a comprehensive analysis and discussion of the risk between diverse NMDs and CAD by utilizing the data derived from NHIRD. The findings of the study indicate that conducting such an analysis would be arduous to accomplish by conventional methods.

### 4.3. Contributions and Limitations

Our study contributes in three significant ways. Firstly, the study’s validity is enhanced by the utilization of a large, representative nationwide database, which provides a population-based assessment of CAD risk in individuals with prior CTS and other NMD diagnoses. Our article employs diverse analytical methodologies, revealing that, whether adopting a retrospective case–control study design or a prospective cohort study design, there exists a discernible correlation between CTS and CAD. This concurrent observation serves to fortify the robustness and persuasive quality of the analytical outcomes, a facet seldom exploited in analogous numerical analysis articles. Secondly, we retrospectively examined NHIRD records to analyze disease time sequences and levels, investigating potential causality between CTS and CAD. Our findings revealed an elevated incidence of CAD in the case group with a previous CTS diagnosis. In most medical aspects, compared to CTS, which could be attributed to functional level disease, CAD is a much more severe and complicated disorder with substantial damage and atherosclerosis changes in the coronary vessels and pericardium circulation system; then, there may be serious time-related casualty in the development of CTS into CAD. After all, in general clinical applications and under some conditions, it is difficult to clearly observe the possible corresponding changes over a long period of time for a single case or a specific disease group; the NHIRD study is a relatively applicable research model. Thirdly, it should be noted that our study is the first to examine the relationship between CAD and various NMDs for discussion purposes. By comparing the results to those obtained for ST and ulnar side NMDs, we aim to shed light on the potential association between CTS and CAD.

There are still several limitations to this research, and some of the elements were based on the restrictions of the NHIRD database itself. First, the medical database of NHIRD for analysis did not record the severity of or variation in the diseases, such as unilateral or bilateral CTS. Secondly, although HTN, DM, and hyperlipidemia were listed as comorbidities of CAD for analysis, the drug history for these chronic diseases may also affect the stiffness of vessels [48,49]. For example, the information not registered in NHIRD, such as the self-administration of related drugs, may cause an increase in bias during analysis. Similarly, some of the risk factors, including smoking and alcohol history, occupational factors, particularly vibrating tools and those demanding a strong hand grip [50], lifestyle living habits, BMI, and hereditary conditions that could affect CAD or CTS, are not available for analysis. Specifically, not all of the risk factors associated with CAD, CTS, and NMDs were included. The disadvantages for most been challenged in NHIRD data analysis is only to figure out the main trend and possibility. Third, there are numerous NMDs remaining that are not included in the study; for our purposes, it is appropriate to speculate on the NMDs in the nearby region of the forearm rather than to bring in all of them to analyze the relationship to CAD.

Finally, although in the discussion paragraph of this article we provide some possible mechanisms in the literature and empirical medical data as evidence for the association between CTS and CAD, this study still cannot explain why this association has not been seen in other NMDs diseases. This is also an area that needs more relevant research in the future. Furthermore, our data indicated an association between CTS, other NMDs, and CAD over a 10-year period; how to realize the variety of the association over a longer time period remains unknown. Moreover, the database of analysis in the study is confined to the population of Taiwan, and it is uncertain that the NHIRD records and results could be representative of other regions or global population characteristics. As for the interventions into CTS subjects to alleviate the prevalence of CAD, more advanced and rigorous research with long observation periods and objective evaluation methods are needed.

## 5. Conclusions

The CAD patients were more likely to have a previous diagnosis of CTS. However, apart from CTS, there is no such significant association between CAD and other NMDs, including stenosing tenosynovitis or medial epicondylitis/ulnar nerve lesion/ulnar nerve injury. These results indicated that median nerve injury or CTS may uniquely serve as a predisposing factor of CAD rather than the other NMDs of the forearm. In future, the pathological connection and possible mechanism of CTS and CAD deserve a more in-depth study.

## Figures and Tables

**Table 1:** Demographics and clinical characteristics of the study population.

Variable	No CAD (Controls)		CAD (Cases)		*p*-Value
	(*n* = 64,025)		(*n* = 64,025)		
	*n*	%	*n*	%	
**Sex ^Ɨ^**					------
Female	30,724	47.99	30,724	47.99	
Male	33,301	52.01	33,301	52.01	
**Age, years ^Ɨ^**					------
<18	210	0.33	210	0.33	
18–39	3516	5.49	3516	5.49	
40–65	26,414	41.26	26,414	41.26	
>65	33,885	52.92	33,885	52.92	
**Risk factors ^Ɨ^**					
CTS	832	1.3	1454	2.27	<0.0001 ***
Stenosing tenosynovitis	76	0.12	92	0.14	0.2188
Ulnar side NMDs (1 + 2 + 3)	134	0.21	183	0.29	0.0065 **
^1^ Medial epicondylitis	57	0.09	56	0.09	0.925
^2^ Lesion of the ulnar nerve	71	0.11	115	0.18	0.0014 **
^3^ Ulnar nerve injury	6	0.01	17	0.03	0.0285 *
**Comorbidity ^Ɨ^**					
DM	8337	13.02	16,926	26.44	<0.0001 ***
Hypertension	19,542	30.52	41,742	65.2	<0.0001 ***
Hyperlipidemia	10,263	16.03	23,796	37.17	<0.0001 ***

CAD = coronary artery disease; CTS = carpel tunnel syndrome; NMDs = neuromuscular disorders; DM = diabetes mellitus. Tests used: ^Ɨ^ Pearson’s chi-squared test. * *p* < 0.05; ** *p* < 0.01; *** *p* < 0.001 vs. controls.

**Table 2:** Odds ratios (ORs) and 95% confidence intervals (CIs) of univariate and multivariate logistic regression analyses using patients’ characteristics as predictors of the development of coronary artery disease (CAD) and/or carpal tunnel syndrome (CTS), stenosing tenosynovitis, and ulnar side neuromuscular disorders (NMDs).

Variable	Crude			Adjusted		
	ORs	(95% CI)	*p*-Value	ORs ^§^	(95% CI)	*p*-Value
**Sex**						
Female	1.00			1.00		
Male	1.00	(0.98–1.02)	0.99	1.10	(1.07–1.12)	<0.0001 ***
**Age, years**						
<18	1.00			1.00		
18–39	1.00	(0.82–1.22)	0.99	0.77	(0.63–0.94)	0.0118 *
40–65	1.00	(0.83–1.21)	0.99	0.51	(0.42–0.62)	<0.0001 ***
>65	1.00	(0.83–1.21)	0.99	0.33	(0.27–0.40)	<0.0001 ***
**Risk factors ^Ɨ^ (ref = non-)**						
CTS	1.77	(1.62–1.92)	<0.0001 ***	1.45	(1.32–1.59)	<0.0001 ***
Stenosing tenosynovitis	1.21	(0.89–1.64)	0.2188	1.12	(0.8–1.55)	0.5195
Ulnar side NMDs	1.36	(1.09–1.70)	0.0065 **	1.10	(0.87–1.41)	0.4277
**Comorbidity ^Ɨ^ (ref = non-)**						
DM	2.40	(2.33–2.47)	<0.0001 ***	1.36	(1.31–1.40)	<0.0001 ***
Hypertension	4.26	(4.16–4.36)	<0.0001 ***	4.13	(4.02–4.24)	<0.0001 ***
Hyperlipidemia	3.10	(3.02–3.18)	<0.0001 ***	2.05	(1.99–2.11)	<0.0001 ***

^§^ Adjusted for CTS, stenosing tenosynovitis, age, ulnar side NMDs (medial epicondylitis or lesion of the ulnar nerve or ulnar nerve injury included), sex, age, DM, hypertension, and hyperlipidemia. ^Ɨ^ The reference population consisted of patients without the comorbidity. NMDs = neuromuscular disorders; DM = diabetes mellitus. * *p* < 0.05; ** *p* < 0.01; *** *p* < 0.001 vs. reference population.

**Table 3:** Incidence rates, hazard ratio, and confidence intervals of coronary artery disease (CAD) association with/without carpal tunnel syndrome (CTS) in the stratification of sex, age, and comorbidity.

Variables	Carpel Tunnel Syndrome						Compared with Non-CTS Group	
	Non-CTS Group			CTS Group			Crude HR	Adjusted HR
	Control Group (N = 6188)			Cohort Group (N = 6188)				
	Event	Person Years	IR †	Event	Person Years	IR †	(95% CI)	(95% CI)
**Total**	282	49,081	5.75	508	48,265	10.53	1.83 (1.58–2.12) ***	1.61 (1.39–1.87) ***
**Sex**								
Female	210	37,005	5.67	366	36,472	10.04	1.77 (1.49–2.09) ***	1.59 (1.34–1.89) ***
Male	72	12,076	5.96	142	11,793	12.04	2.02 (1.52–2.68) ***	1.68 (1.26–2.23) ***
**Age, years**								
<20	0	346	0	0	343	0	-	-
20–39	16	13,675	1.17	49	13,691	3.58	3.05 (1.74–5.36) ***	2.35 (1.32–4.21) **
40–59	211	31,518	6.69	380	30,730	12.37	1.85 (1.56–2.18) ***	1.6 (1.35–1.9) ***
>60	55	3542	15.53	79	3501	22.56	1.46 (1.03–2.05) *	1.35 (0.96–1.92)
**Baseline comorbidity**								
**DM**								
No	235	46,217	5.08	408	44,193	9.23	1.82 (1.55–2.13) ***	1.6 (1.36–1.88) ***
Yes	47	2864	16.41	100	4072	24.56	1.5 (1.06–2.12) *	1.54 (1.08–2.18) *
**HTN**								
No	179	43,133	4.15	295	39,795	7.41	1.79 (1.48–2.15) ***	1.66 (1.37–2) ***
Yes	103	5948	17.32	213	8470	25.15	1.45 (1.15–1.84) **	1.45 (1.14–1.83) **
**Hyperlipidemia**								
No	203	43,981	4.62	308	39,417	7.81	1.69 (1.42–2.02) ***	1.58 (1.32–1.89) ***
Yes	79	5100	15.49	200	8848	22.61	1.46 (1.13–1.9) **	1.52 (1.17–1.98) **

IR † = incidence rates, per 1000 person-years; HR = hazard ratio; CI = confidence interval; CTS = carpel tunnel syndrome; DM = diabetes mellitus. Adjusted HR: adjusted for carpel tunnel syndrome, age, gender, hypertension, hyperlipidemia, and DM in Cox proportional hazards regression * *p* < 0.05; ** *p* < 0.01; *** *p* < 0.001.

## Data Availability

The NHIRD data used to support the findings of this study are included within the article.
